# Molecular Tumor Boards: Ethical Issues in the New Era of Data Medicine

**DOI:** 10.1007/s11948-017-9880-8

**Published:** 2017-03-09

**Authors:** Henri-Corto Stoeklé, Marie-France Mamzer-Bruneel, Charles-Henry Frouart, Christophe Le Tourneau, Pierre Laurent-Puig, Guillaume Vogt, Christian Hervé

**Affiliations:** 1 Medical Ethics and Legal Medicine Laboratory EA4569, Paris Descartes University, Centre Universitaire des Saints-Pères, Paris, France; 2 Cancer Research for Personalized Medicine (CARPEM), Paris Descartes, APHP (HEGP, Cochin, Necker) INSERM, Paris, France; 3 Neglected Human Genetics, Centre National de Genotypage (CNG–CEA), Evry, France; 4 Assistance Publique-Hôpitaux de Paris AP-HP, Necker-Enfants Malades Hospital, Paris, France; 5 Inserm UMR-S1147, Paris Descartes University, Centre Universitaire des Saints-Pères, Paris, France; 6 Department of Medical Oncology, Institut Curie, Paris, Saint-Cloud, France; 7 EA7285, Versailles University, Saint-Quentin-en-Yvelines, Versailles, France

**Keywords:** Personalized medicine, Data medicine (DM), Molecular tumor board (MTB), Electronic informed consent (e-IC), Dynamic consent, Biobank, Database, Genomic, Bioinformatic, Ethical issues

## Abstract

The practice and development of modern medicine requires large amounts of data, particularly in the domain of cancer. The future of personalized medicine lies neither with “genomic medicine” nor with “precision medicine”, but with “data medicine” (DM) (big data, data mining). The establishment of this DM has required far-reaching changes, to establish four essential elements connecting patients and doctors: biobanks, databases, bioinformatic platforms and genomic platforms. The “transformation” of scientific research areas, such as genetics, bioinformatics and biostatistics, into clinical specialties has generated a new vision of care. Molecular tumor boards (MTB) are one response to these changes and are now providing better access to next-generation sequencing (NGS) and new cancer treatments to patients with inoperable or metastatic cancers, and those for whom the usual treatment has failed. However, MTB face a crucial ethical challenge: maintaining and improving the trust of patients, clinicians, researchers and industry in academic medical centers supported by private or public funding rather than providing genetic data directly to private companies. We believe that, in this era of DM, appropriate modern digital communication networks will be required to maintain this trust and to improve the organization and effectiveness of the system. There is, therefore, a need to reconsider the form and content of informed consent (IC) documents at all academic medical centers and to introduce dynamic and electronic informed consent (e-IC).

## Introduction

In 1950, the American mathematician Norbert Wiener proposed that “*Society can only be understood through a study of the messages and the communication facilities which belong to it*” (Weiner 1950). Extrapolating this idea to medicine, we can see that the communications facilities used are increasingly electronic and that the messages transmitted contain ever-larger amounts of genetic data from patients for use in care or research. Medicine now requires large amounts of data to function and develop, particularly in the domain of cancer. This is why, in our opinion, personalized medicine should not be called “genomic medicine” or “precision medicine”, but “data medicine” (DM) (although this term is currently rarely used in the scientific literature).

Indeed, advances in computer science and technology are leading to new clinical tools and methods (specific algorithms) for deducing suspected clinical information (e.g. targeted mutations approved) from a large amount of raw data, including genetic data in particular. This data-mining approach (Delort 2015) would appear at first glance to fit into a context of “genomic medicine” or “precision medicine”. However, some of these new tools and methods (e.g. machine or deep learning) are making it possible to induce unsuspected clinical information (e.g. new mutation or validation of a targeted mutation non-approved) from a large amount of raw data (possibly even larger amounts of even more preliminary data). The big data approach (Delort 2015). DM can therefore be seen as more complex than genomic or precision medicine and encompassing both the “old” *data mining* approach and the “new” *big data* approach. This is a particularly important point that needs to be taken on board by all.

**Fig. 1:**
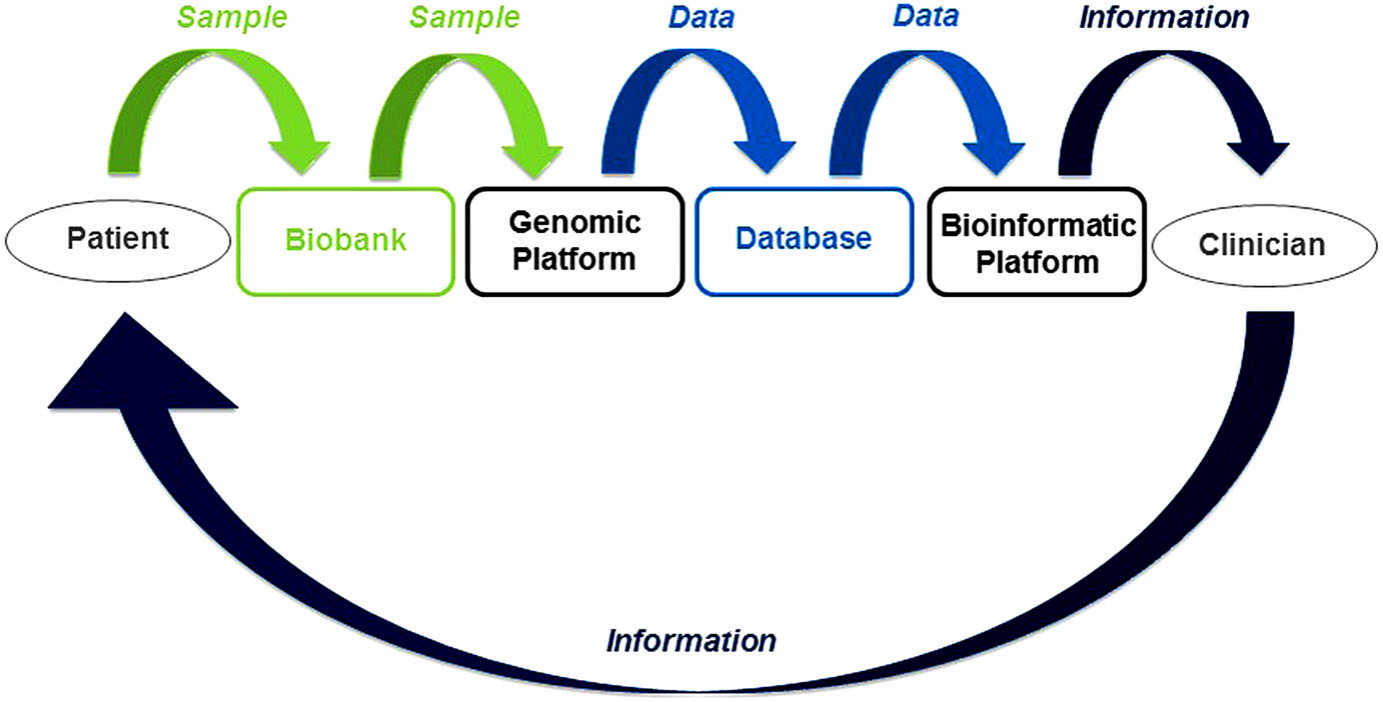
Model of the data medicine process. Biological sample flows and biobanks are shown in *green*. Data flows and databases are shown in *light blue*. Information flows are shown in *dark blue*

In this new framework, DM has required the establishment of a set of four essential elements connecting patients and clinicians (Fig. 1): (1) biobanks for the storage of biological samples; (2) genomics platforms, for generating genetic data from biological samples; (3) databases, for the storage of genetic data; (4) bioinformatics platforms, for the production of clinical information from genetic data (and other raw data). The “transformation” of scientific research areas, such as genetics, bioinformatics and biostatistics, into clinical specialties has led to the emergence of a new vision of care, mostly due to demonstrated clinical benefits of DM and the significant and rapid decrease in the price of new technologies.

At most academic medical centers, the meetings at which cancer diagnosis and treatment are discussed, known as tumor boards (TB) are open only to oncologists, surgeons, pathologists and radiologists (Kuroki et al. 2010). However, some patients have inoperable tumors and others suffer treatment failure after surgery. This has led to the emergence of a new type of structure, molecular tumor boards (MTB), in which surgeons are replaced by geneticists, bioinformaticians and biostatisticians (Schwaederle et al. 2014). These structures are endowed with a new mixture of skills, to meet new challenges in cancer care and treatment.

MTB are providing better access to next-generation sequencing (NGS) and new cancer treatments to patients with inoperable or metastatic tumors, and patients for whom the usual treatment has failed, increasing the chances of these patients living longer or even avoiding a fatal outcome of their disease (Erdmann 2015). Before the creation of MTB, translational research was established to provide researchers and industry with better access to patients, biological samples and genetic data via academic medical centers. Some MTB are thus located at centers of translational research (Kamal et al. 2015).

The equivalence of public and private structures is complex, and a first, key question concerns the status of the genetic data generated. Are they genetic data for care, for research, or for both at the same time? This question may seem trivial, but the answer is crucial, for both patient autonomy and for the chain of trust connecting patients, clinicians, researchers and industry in real time, at academic medical centers, in the new era of DM. Clinical trials and retrospective studies are increasingly frequent in this new era, but they remain poorly understood by patients (Jefford and Moore 2008).

This lack of understanding is behind one of the most important “ethical challenges” confronting MTB: finding ways to maintain and improve the trust of patients, clinicians, researchers and industrials in academic medical centers in the era of DM. Based on Weiner’s ideas, we believe that modern and appropriate facilities (electronic and dynamic) for communication between the varies parties and clear messages (purpose of care or research) will be required to achieve this goal. (Annas 2001; Dixon et al. 2014; Kaye et al. 2015; Williams et al. 2015; Spencer et al. 2016; Steinsbekk et al. 2013). We think that the form and content of informed consent (IC) documents should be reconsidered, in the Internet age, when next-generation sequencing may be carried out for any patient in a research protocol or receiving care.

We think that patients would benefit from greater honesty, and transparency, but also from modern approaches and real-time interactions concerning the use of their biological samples and genetic data, to encourage them to continue going to academic medical centers rather than providing genetic data directly to private companies, as has been observed in the recent past (Stoekle et al. 2016). Several new private companies specializing in genomics or informatics have recently been set up in the US and are establishing strong communication networks in real time, directly between patients and the pharmaceutical industry (two-sided market) via Internet and cloud computing, without the involvement of academic medical centers (Stoekle et al. 2016). These new issues are particularly important in countries in which social insurance systems are mostly based on solidarity (social security) rather than individual ability to pay. Patients may thus, one day, have free access to their own genetic information, which they will be able to use as they see fit.

A global view is essential, to determine how MTB can establish a win–win strategy with patients that goes beyond current patient-physician relationships. We discuss here the social, scientific, economic and ethical issues raised by new dynamic and interactive relationships between patients, clinicians, researchers and industry that could serve as the basis of DM at an academic medical center.

## The Molecular Tumor Board Model

**Fig. 2:**
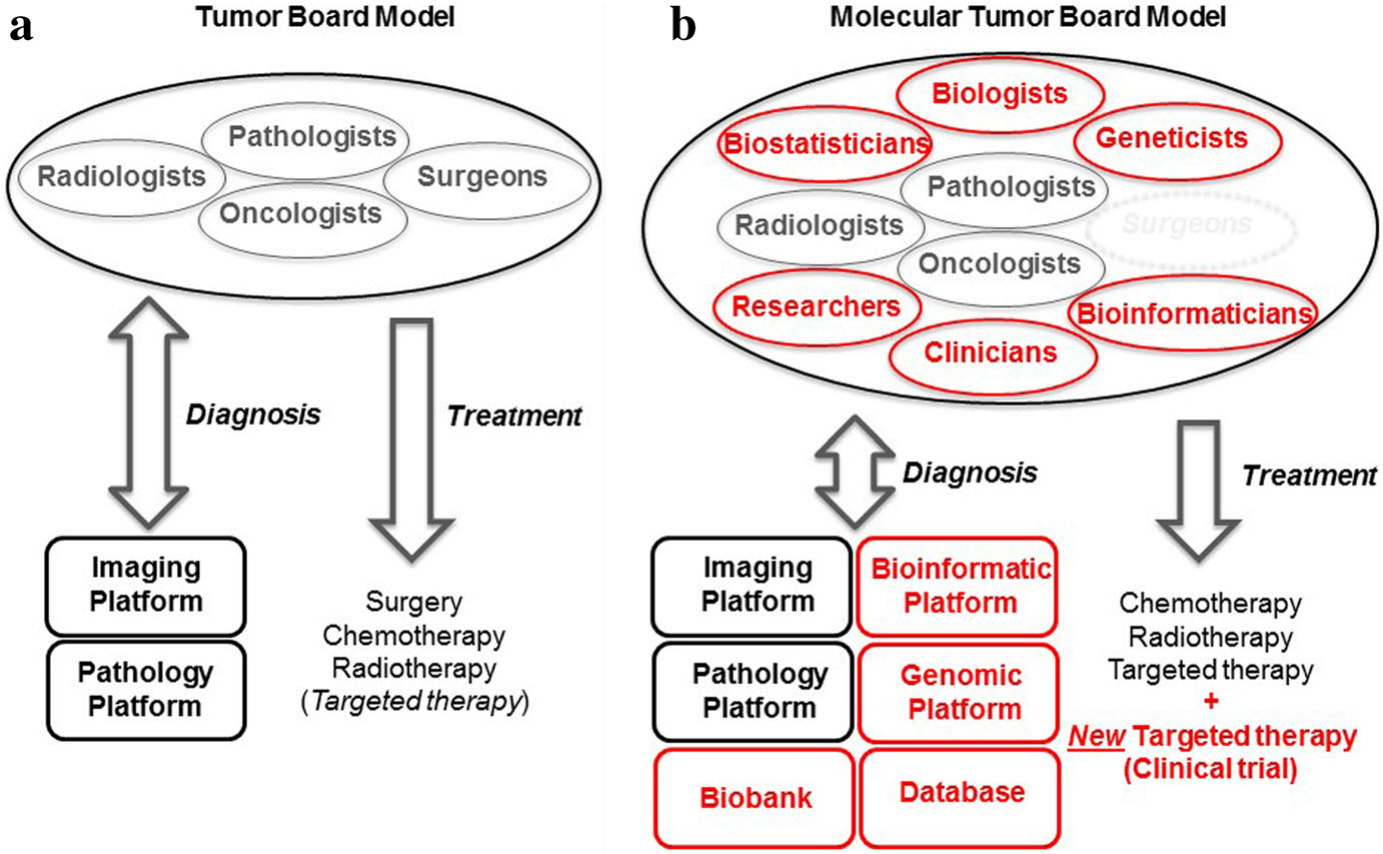
**a** The tumor board model, versus **b** the molecular tumor board model

Tumor boards (TB) (or “multidisciplinary teams” (MDT) in the UK, Commonwealth and Switzerland) have long been a feature in scientific studies (Gross 1987; Wright et al. 2007; Coory et al. 2008; Fleissig et al. 2006). According to the National Institutes of Health (NIH) in the US, TBs are “*a treatment planning approach in which a number of doctors who are experts in different specialties (disciplines) review and discuss the medical condition and treatment options of patient*”. Three different specialties are, therefore, generally represented: a medical oncologist, a surgical oncologist and a radiation oncologist [NIH website (www.cancer.gov)]. The TB may also include a pathologist, although this possibility is not discussed by the NIH (Fig. 2a) (Kuroki et al. 2010).

The primary objective of TB is to improve diagnosis and treatment selection, thereby improving care management for the patient (Fig. 2a) (Keating et al. 2013). However, TB were also designed to improve communication and interactions between the different specialties (e.g. pathology and imaging platforms) (Ruhstaller et al. 2006). However, the specialties represented on the TB are essentially clinical. There are no scientists among TB members and little communication occurs between clinicians and scientists (Gross 1987; Wright et al. 2007; Coory et al. 2008; Fleissig et al. 2006; Keating et al. 2013; Ruhstaller et al. 2006). This absence of scientists may reflect the predominance of histological, tissue- or organ-based approaches. In such conditions, scientific expertise from a genomics or bioinformatics platform is not required for diagnosis (radiology, immunochemistry) or for the selection of the “usual” types of treatment (surgery, chemotherapy, radiotherapy) (Fig. 2a).

In parallel, scientists have been involved in clinical research, creating new cancer treatments (targeted therapies), such as Herceptin^®^ (trastuzumab) and Avastin^®^ (bevacizumab), which were developed by the American biotechnology company Genentech, Inc. (Roche) and have been on sale since the 1990s. Formally, TB were not designed to provide patients with support after surgery, but some patients have inoperable tumors or suffer treatment failure after surgery; others have metastatic tumors or suffer treatment failure on usual treatments (Erdmann 2015). The advent of these targeted therapies has, thus, opened up new possible roles for scientists in the domain of treatment, particularly for patients with otherwise unresponsive tumors (Santa-Maria and Gradishar 2015). Since the advent of targeted therapy, cancer can be seen not only as the consequence of an organ dysfunction, but also as a molecular abnormality due to a specific genetic alteration that must be identified by molecular tests, such as DNA sequencing, in particular.

The choice of name—“molecular tumor board” (MTB)—perfectly reflects this “scientization” of medicine (Vanneman and Dranoff 2012; Sawyers 2004; Rouviere et al. 2015; Huang et al. 2014; Dausset 1996). For the moment, very few scientific papers have referred to a MTB. Only the University of California San Diego (UCSD) Moores Cancer Center (US) (Schwaederle et al. 2014), the Cleveland Clinic (US) (Sohal et al. 2016) and the Curie Institute (France) (Kamal et al. 2015) seem to have already published research articles or reviews about a MTB or similar structures with a different name. Indeed, as for TB, MTB may also be given other names, such as “molecular biology boards” (Curie Institute) or “genomics tumor boards” (Cleveland Clinic). An article published in *Nature Medicine* in July 2015 estimated that there were 30 MTB across the US (Erdmann 2015).

In France, MTB are called *réunion de concertation pluridisciplinaire moléculaire* (molecular multidisciplinary meetings). They have been developed at the Curie Institute (Kamal et al. 2015), the Gustave Roussy Institute (Gillet 2010) and the *Assistance Publique des Hôpitaux de Paris* (the Parisian hospital network), including, in particular, *Hôpital Européen Georges Pompidou* (Georges Pompidou European Hospital) and *Hôpital Tenon* (Tenon Hospital) (Rouviere et al. 2015). Other such structures are likely to develop throughout France in the near future, in response to new health and research policies concerning cancer diagnosis and treatment.

Regardless of differences in the names of these structures between countries, their organization and function appear to be similar in the US and France: a group of oncologists, radiologists, pathologists, biologists, geneticists, bioinformaticians, biostatisticians, researchers and clinicians providing patients presenting treatment failure with access to NGS diagnostic techniques (genomics and bioinformatics platforms) and new cancer treatments (targeted therapies), mostly through inclusion in clinical trials. Surgery has been replaced in these structures by domains of scientific research, such as genetics, bioinformatics and biostatistics (Fig. 2b), which are developing into new medical specialties, due to demonstrated clinical benefits and a rapid and significant fall in the cost of new technologies, such as NGS.

For 30 years, all DNA sequencing was carried out by the Sanger method (Schuster 2008). NGS devices first appeared in 2007 and owe their success to synchronous sequence analysis, resulting in faster, more sensitive analyses, at a lower overall cost (Meldrum et al. 2011). Indeed, whereas Sanger’s direct (or first-generation) sequencing method requires the generation of DNA strands of different lengths labeled with a fluorophore for analysis, NGS methods reconstruct previously prepared DNA strands by directly determining the nucleic acids incorporated (Shendure and Ji 2008). NGS costs have decreased markedly, and this method is now being transferred from purely research uses to clinical applications. Diagnostic NGS (RNA, gene panels and whole-exome sequencing) is specific to MTB and helps to provide patients with access to targeted therapy (Erdmann 2015). There are currently moves in France to provide full social security reimbursement for patients undergoing NGS tests.

According to the NIH website (NIH website (www.cancer.gov)), targeted therapy (also known as “molecularly targeted drugs”, “molecularly targeted therapy”, or “precision medicine”) is more qualitative in its action, whereas chemotherapy is more quantitative: targeted therapy acts on specific molecular targets in tumor cells, whereas chemotherapy acts on all rapidly dividing cells (normal and cancerous); targeted therapies often block tumor cell proliferation (cytostatic), whereas chemotherapy kills tumor cells (cytotoxic). The available targeted therapies include hormone therapy, signal transduction inhibitors, gene expression modulators, apoptosis inducers, angiogenesis inhibitors, immunotherapies and toxin delivery molecules (Sawyers 2004; Huang et al. 2014).

The FDA has approved some of these therapies, which have clearly proved effective against certain types of cancer (Herceptin^®^ in breast cancer), but most are still at the clinical trial stage. This is not particularly surprising, because these treatments are in their infancy, and some have been approved for the treatment of one type of cancer but may be useful against others and, therefore, require further trials to gain approval for an expansion of their indications. Most of the patients undergoing sequencing do not receive targeted treatment, even in trials. According to a USCD study, “*the most common reasons for being unable to act on molecular diagnostic results were that patients were ineligible for or could not take part in an appropriately targeted trial and/or that insurance would not cover the cognate agents”* (Schwaederle et al. 2014). A similar observation was made at the Cleveland Clinic, where a study revealed that only 24 of the 250 patients with selected solid tumors received targeted therapy, mostly due to a lack of clinical access to treatment or a deterioration of the patient’s clinical condition (Sohal et al. 2016).

## Ethical Issues

**Fig. 3:**
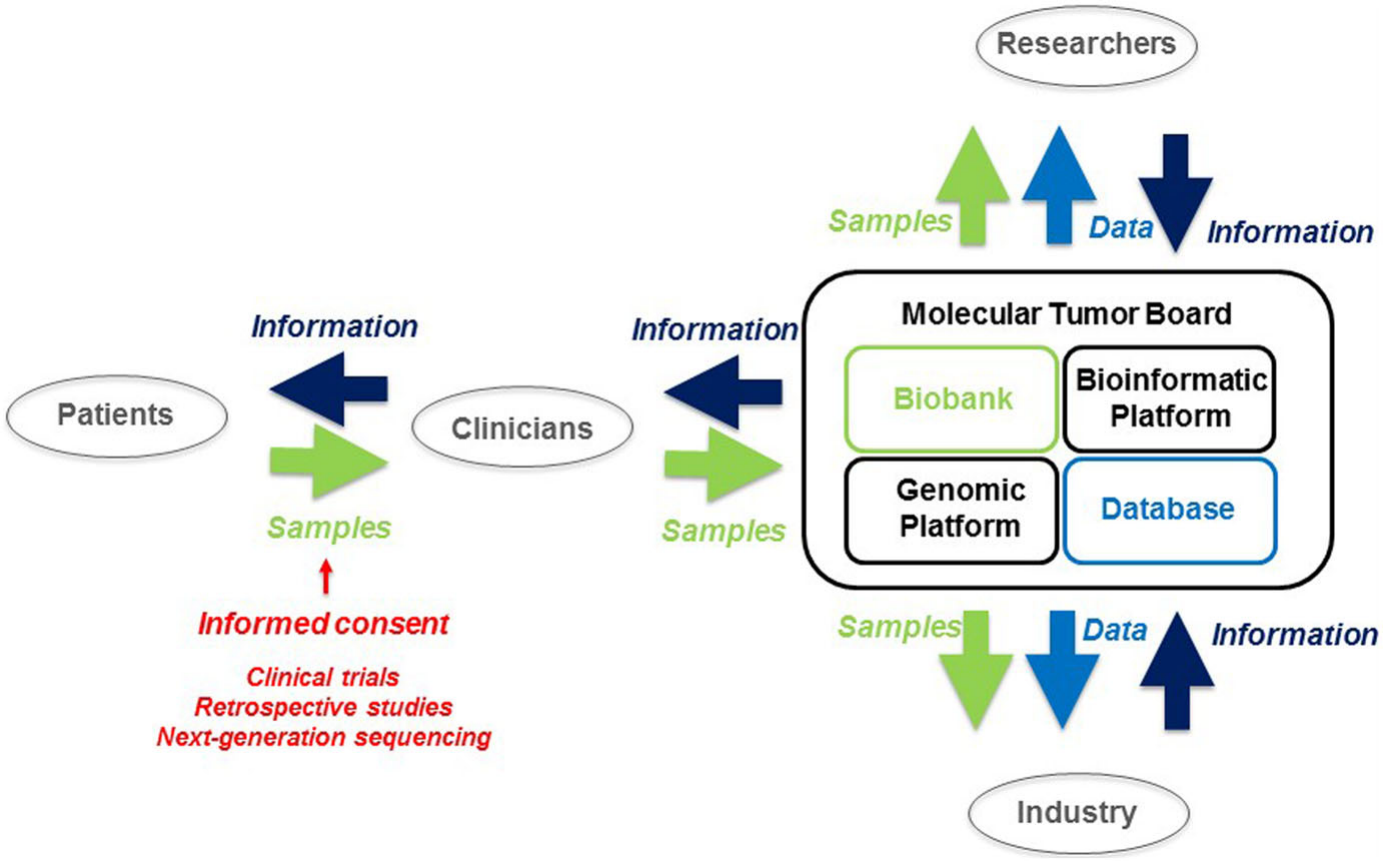
Model of the molecular tumor board process. Biological sample flows and biobanks are shown in *green.* Data flows and databases are shown in *light blue*. Information flows are shown in *dark blue*

According the previous publications, independently of their clinical functions (which form the focus of most studies), MTB are also starting to coordinate the flow of biological samples, genetic data and information between patients and clinicians, but also between researchers at academic medical centers and industry, through clinical trials and retrospective studies (Fig. 3). If the intensity of these flows continues to increase, then, in the future, MTB or similar structures may play a much greater role in deciding who has access to these data. Thus, the MTB or similar structures may become major decision-makers at academic medical centers, facilitating the transfer of data from care to research contexts.

This would also improve the tracing of important decision-making processes, and their organization around a group of field experts in DM. In this era of major revolutions in science and technology (big data, data mining, machine learning, artificial intelligence, cloud computing, etc.), it has been suggested that the experts involved in these decision-making processes will no longer be exclusively medical doctors. Instead, these groups of experts will include scientists, statisticians and informaticians. These changes are likely to have a strong impact, and they raise several ethical issues, some of which are already of importance, including the reasons for communicating with patients and how best to do so, particularly as concerns the biobanks, databases, genomic or bioinformatic processes, clinical trials and retrospective studies developed through these flows. They also add to the risk of unequal access to these technologies on the basis of socioeconomic or genetic criteria.

Many clinical trials may be considered as treatment options because they provide some patients with a “last chance” of recovery. Nevertheless, clinical care and clinical research differ in that the objective of care is to be of benefit to a patient, whereas the objective of research is to gain new knowledge (Jefford and Moore 2008). The information provided to the patient when consent is sought also differs considerably between clinical care and research: for research, the information and the goals are explained in the conditional tense, because there are far fewer certainties than for clinical care. Indeed, one of the first objectives of clinical trials is to determine whether the new treatment works better than the old one. Furthermore, the new treatment may entail unknown or theoretical risks, which is less likely to be the case for a treatment that is already authorized. Thus, the first ethical issue raised by a MTB is the following: if a treatment is prescribed on inclusion in a clinical trial, can it still be considered to be clinical care?

This is a particularly important issue, given that scientific studies of a MTB have reported essentially inclusions in clinical trials rather than the prescription of authorized cancer treatments (Le Tourneau et al. 2012; Kamal et al. 2015; Sohal et al. 2016; Schwaederle et al. 2014). Furthermore, there are several different levels of clinical trials. For example, phase I clinical trials are designed to assess the toxicity of a new treatment in human subjects (acceptable dose levels) and are often conducted on patients presenting treatment failure (Brown et al. 2011). Therapeutic benefits are more likely to be seen in phase II and III trials, which test therapeutic efficacy rather than toxicity. However, the issue is more complex for targeted cancer therapy, and several studies have reported direct therapeutic benefit in phase I trials (Khan et al. 2016), even though the patient’s clinical condition (Sohal et al. 2016) and the quality or quantity of biopsy material can be a barrier to participation in such clinical trials (Lim et al. 2016).

For these reasons, “possible care”, “conditional care” or “potential care” should be considered, rather than just “care”, and these concepts should be explained in more detail to patients to ensure that they understand the consent form they are asked to sign. These adjectives qualifying “care” are therefore of great importance, because a short-term individual benefit is for the moment, merely possible; in theory, trials are seeking to obtain long-term collective benefit. But can this “care modify the “theory”? Perhaps, and it might even be a good thing if it did, because the frequent inclusion of cancer patients in clinical trials necessitates a new strategy for communication between patients, clinicians, researchers and industry. We need to make use of ethical discussions about the content and form of IC, to find new ways of improving the patients’ understanding of their participation in clinical trials (Kelley et al. 2015; Kao et al. 2016) (Fig. 3).

The publication of the first draft sequence of the human genome (Lander et al. 2001; Venter et al. 2001), and its completion by the Human Genome Project marked an increase in the strategic importance of DNA banking and data collection. In the past, biological samples were stored in a single laboratory, but large collections of DNA samples are becoming increasingly common in human genetics (Thornton et al. 2005). DNA can be obtained from a number of potential sources, including the blood, the cell and tissue banks of hospitals and academic research centers, and it has been estimated that there are already several hundred million biological samples stored in such repositories (Swede et al. 2007).

Biobanks are becoming increasingly important for the establishment of research infrastructures (Castillo-Pelayo et al. 2015), and the systematization of NGS, clinical trials and retrospective studies in cancer may render these repositories of even greater importance, through a cyclic phenomenon. As more clinical trials are set up and more patients have their DNA sequenced, there will be more biological samples, genetic data and clinical information stored in biobanks, leading to more retrospective studies being carried out and more clinical trials being designed.

Retrospective studies could play a key role in completing the loop. However, researchers cannot know in advance which topics they will address through retrospective research studies. It is not, therefore, possible to provide patients with “perfect information”, that is to say, a description of all possible research purposes in advance, when they sign an informed consent form. Institutional review boards and ethics committees generally require consent to disclose all of the ways in which samples and data may be used and to ensure an acceptable balance between risks and benefits (Godard et al. 2003; Rothstein 2002). This is why some recent studies have tried to determine whether patients prefer a “re-consent” or a “broad-consent” process, but no clear consensus has yet to emerge (Edwards et al. 2016; Goodman et al. 2016).

The increasing number of retrospective studies being carried out on cancer, using data collected in clinical studies, requires a new strategy for communication between patients, clinicians, researchers and industry, and ethical reflections are required about the content and nature of the informed consent collected, with the possibility of returning to patients if necessary (Edwards et al. 2016). This approach should improve the patients’ understanding of research protocols, increasing their autonomy and their trust in academic medical centers (Fig. 3). However, it will require a particularly well-adapted form of informed consent.

Informed consent is much more than a simple form of communication between patients, clinicians, researchers and industry (Annas 2001). With the increasing numbers of retrospective studies based on data from clinical trials in the field of cancer, we need to consider the form of informed consent given, and not just its content, at all academic medical centers at which there are close ties between care and research. Recent innovations in information technology (IT) could drive changes in strategy concerning communication between patients, clinicians, researchers and industry (Kaye et al. 2015).

Trust is based primarily on communication. If patients feel that they can communicate freely, anywhere, at any time and in complete safety, they are more likely to trust the system and to be willing to share their data and biological samples. An electronic informed consent (e-IC), or dynamic consent (Kaye et al. 2015; Steinsbekk et al. 2013; Dixon et al. 2014; Williams et al. 2015; Spencer et al. 2016) system would open up new possibilities: (1) for patients to determine whether or not to give consent, at the time and place of their choosing, (2) for clinicians to ensure, in real time, that a particular piece of information has been transmitted to a particular patient and to determine whether or not the patient concerned has given consent and (3) for researchers and industry, to facilitate the use, in real time, of particular samples or data (Karlson et al. 2016).

The FDA [according to a draft document available from the FDA website (www.fda.gov)] has identified IT as a good way of improving communication between patients and clinicians through multiple media: text, graphics, audio, video, podcasts and interactive websites, biological recognition devices and reader cards to relay information about the study and to obtain informed consent. The FDA draft discusses the possibility of e-IC. Nevertheless, the FDA draft stresses that the content requirements for e-IC should be identical to those for written informed consent: the information should describe and explain clearly the purpose of the study or analysis and it should be easy for the patient to understand the aim of the research.

In general, e-IC facilitates more interactive and dynamic exchanges of information and improves the traceability of biological samples, genetic data and information (Kaye et al. 2015). This process has already been well understood by a number of companies in the US, including Google, Amazon, Facebook, Apple (GAFA), Microsoft, 23andMe, and Helix, a new company that will soon begin trading.

**Fig. 4:**
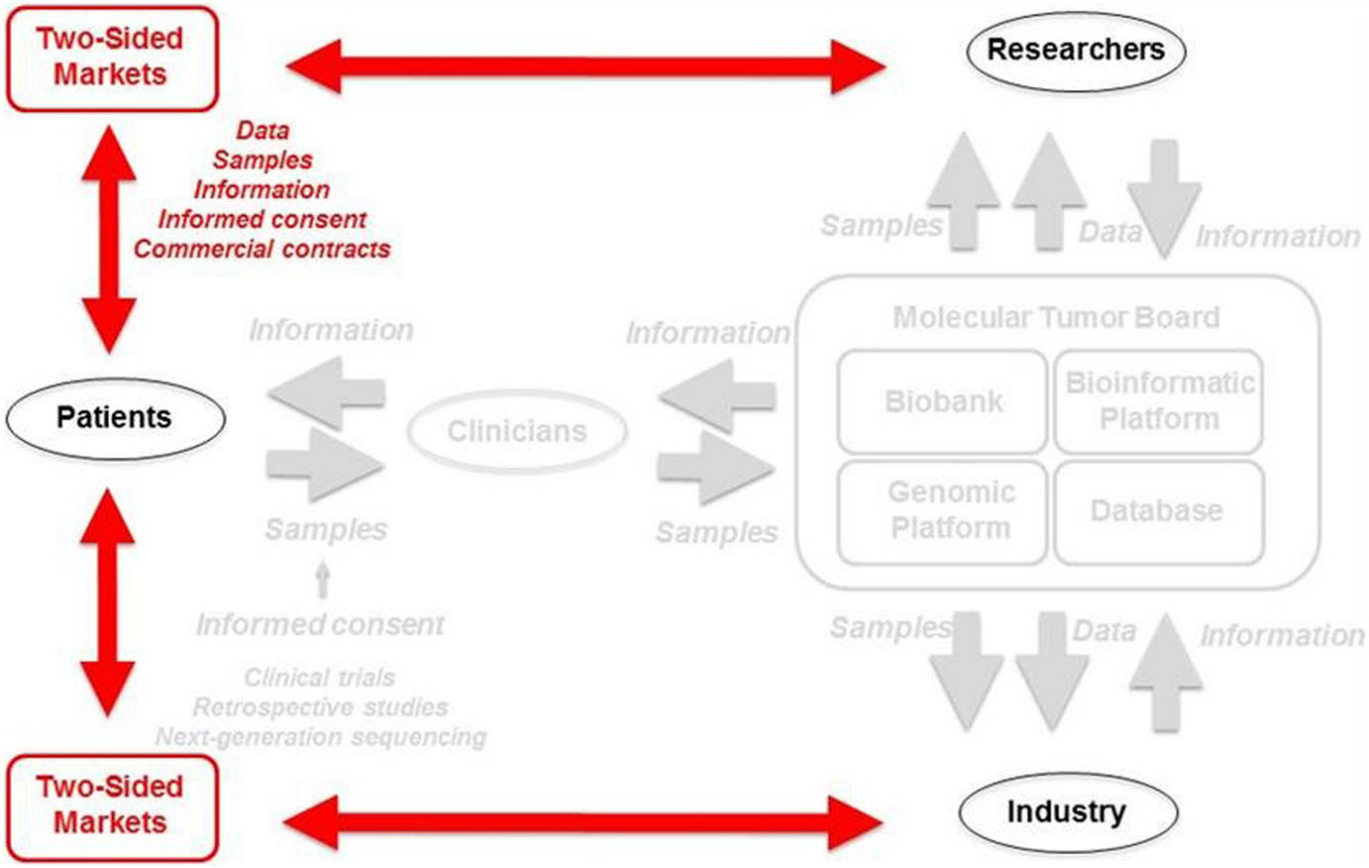
Model of the two-sided market process. New biological sample, data and information flows are shown in *red*

A global view is required to visualize how the rapid sharp decrease in the cost of DNA sequencing and cloud computing has initiated a flow of biological samples and genetic data directly between patients, researchers and industry, leaving some academic medical centers excluded (Fig. 4).

The situation on the ground has changed radically since the 1990s, and several American companies specializing in genomics or informatics appear to have established two-sided market (TSM) platforms through the constitution of biobanks or databases with two kinds of customers: individuals seeking information about their own genes, with or without the need for a prescription from their physicians, and industrial and researcher clients seeking access to the genetic, web behavior and self-reported data obtained from large numbers of people through a commercial contract involving dynamic, electronic informed consent.

23andMe is probably the best known of these companies. Since 2006, this genetic testing company has collected biological samples, genetic data and information from 1,000,000 individuals, at least officially through a direct-to-consumer (DTC) online genetic testing service providing a genetic ancestry report and a genetic health report. In reality, the primary objective of the company was not the provision of DTC testing services, but the establishment of a TSM: promoting itself as providing predictive testing for human genetic diseases and ancestry at a low cost to consumers, whilst establishing a high-value database/biobank for research and industry, for scientific and financial gain (Stoekle et al. 2016). The commercial agreements between 23andMe and Genentech for Parkinson’s disease provide strong evidence of the commercialization of the company’s biobank and database (Mullard 2015).

A new company of the same kind, Helix, seems to be emerging in San Francisco, as reported by the *MIT Technology Review*. This company will collect sputum samples from customers (patients or healthy individuals), who will buy a DNA application for their smartphones. The company will then sequence the exomes of its customers, thereby generating and storing large amounts of genetic information that it will make available to consumers. The entry price for customers is low, at about 100 dollars. This company is based on a TSM model, but it is also a “pay-as-you-go” model (Regalado 2016a). This brings us back to fundamental questions about the content and form of informed consent, but also to questions about the ownership of biological samples and genetic data. Genetic data have become a commodity like any other, at least in North America and some European countries (UK) (Mullard 2015; Dorfman 2013).

However, the ownership of genetic data is not a straightforward issue. First, from an economic point of view, these data must be considered “public goods” (Stiglitz 1999), that is, goods that are both non-rival and non-excludable (Levêque and Ménière 2003). They are non-rival goods because they are not exhaustible: the consumption of public goods by one individual does not prevent another individual from using or consuming the same public goods. They are non-excludable because it is difficult to prevent a particular individual from using them. Once disclosed, genetic data can circulate freely and, in the absence of specific legal provisions, can be used by all, even those not participating in the sequencing or analysis processes.

As a direct consequence of these characteristics, genetic data are not considered to be “scarce” and their ownership cannot be justified on an economic basis, particularly at a time at which the price of sequencing is rapidly decreasing.

On the contrary, it would be appropriate to ensure open access to these data, to prevent their use for research and care being limited to those controlling them. Even if these data cannot be appropriated as such, economic actors try to protect them with legal devices, such as exclusivity agreements, confidentiality agreements or intellectual property rights (Stiglitz 2007–2008), such as those used to protect databases in Europe. Such protective arrangements provide the holders of the data with the power to control downstream markets in care and research (Kitch 1980). This control raises serious ethical problems if it jeopardizes access to care at a reasonable cost for all, or favors a move from social insurance systems based on solidarity to systems based on individual ability to pay.

Other questions are also emerging about the consequences of increases in the flow of genetic data between patients and industry: will industry always need academic medical centers for the development and testing of new drugs and tools? Will patients always need academic medical centers, or even physicians, for diagnosis and treatment? These questions are clearly relevant to the large numbers of individuals subscribing to offers of this kind [>1,000,000 over only 10 years for 23andMe (Stoekle et al. 2016)] and likely to request the new blood test for cancer being developed by Illumina. Indeed, the company recently said that it will form a new company (Grail) to develop a blood test, costing $1000 (or less), for detecting cancers before the onset of symptoms. Illumina predicts that this blood test will reach the market by 2019 (Regalado 2016b).

The Pan Cancer Analysis of Whole Genomes project has demonstrated that the use of cloud computing is much faster and cheaper than the use of conventional academic data centers for the analysis of large amounts of data. In this context, another approach to ensuring free access might be to ensure that all major genetic data be uploaded onto academic and commercial clouds, to prevent private companies, such as GAFA, from obtaining full control over these data (Stein et al. 2015). This process could be managed by MTB and e-IC, reducing costs by keeping the patients out of hospital but staying in real-time communication with them. IT could be used to manage all communication facilities for the development of cancer research, but it would not be competent to manage the message. We believe that academic medical centers remain the best structure for this purpose. This principle seems to have been applied by the Geisinger Health Institute (US) since 2006 [Geisinger website (www.geisinger.org)]. However, the final decision will probably depend on whether societies choose to organize their social insurance systems on the basis of solidarity, as in France, or according to individual ability to pay, as in the United States and many other countries.

## Conclusion

In the new era of DM, biobanking, data banking, integration and processing are becoming essential. It is now clear that clinicians, researchers and industry are starting to understand the need to use genomics, bioinformatics, biostatistics, computer algorithms and machine or deep learning to handle the large amounts of complex genetic data produced and banked effectively for use in care and research in academic medical centers. However, it remains unclear how and why they should provide such data to patients. MTB and e-IC are, thus, much more than just simple structures or forms. They are new ways of organizing the functioning of academic medical centers for DM, to strengthen ties with patients, clinicians, researchers and industry, and to prevent private companies from establishing a monopoly for the storage and analysis of biological samples and genetic data. This issue is of particular importance in countries in which social insurance systems are based on solidarity, rather than individual ability to pay. Yesterday’s world belonged to countries with oil wells and refineries. Tomorrow’s world will belong to those with biobanks, databases, algorithms and artificial intelligence for the generation of big data and data mining. We all need to be aware of this, patients above all.
